# Crowded out

**Published:** 1867-09

**Authors:** 


### *Crowded Out.*

The article by Dr. Hutchins, our Philadelphia correspondent, is unfortunately crowded out of the present number. Will appear next month.

